# Exogenous Coronavirus Interacts With Endogenous Retrotransposon in Human Cells

**DOI:** 10.3389/fcimb.2021.609160

**Published:** 2021-02-25

**Authors:** Ying Yin, Xiao-zhao Liu, Ximiao He, Li-quan Zhou

**Affiliations:** ^1^ Department of Physiology, School of Basic Medicine, Tongji Medical College, Huazhong University of Science and Technology, Wuhan, China; ^2^ Institute of Reproductive Health, Tongji Medical College, Huazhong University of Science and Technology, Wuhan, China; ^3^ Center for Genomics and Proteomics Research, School of Basic Medicine, Tongji Medical College, Huazhong University of Science and Technology, Wuhan, China; ^4^ Hubei Key Laboratory of Drug Target Research and Pharmacodynamic Evaluation, Huazhong University of Science and Technology, Wuhan, China

**Keywords:** coronavirus, retrotransposon, SARS-CoV-2, TET, long interspersed nuclear element

## Abstract

There is an increased global outbreak of diseases caused by coronaviruses affecting respiratory tracts of birds and mammals. Recent dangerous coronaviruses are MERS-CoV, SARS-CoV, and SARS-CoV-2, causing respiratory illness and even failure of several organs. However, profound impact of coronavirus on host cells remains elusive. In this study, we analyzed transcriptome of MERS-CoV, SARS-CoV, and SARS-CoV-2 infected human lung-derived cells, and observed that infection of these coronaviruses all induced increase of retrotransposon expression with upregulation of TET genes. Upregulation of retrotransposon was also observed in SARS-CoV-2 infected human intestinal organoids. Retrotransposon upregulation may lead to increased genome instability and enhanced expression of genes with readthrough from retrotransposons. Therefore, people with higher basal level of retrotransposon such as cancer patients and aged people may have increased risk of symptomatic infection. Additionally, we show evidence supporting long-term epigenetic inheritance of retrotransposon upregulation. We also observed chimeric transcripts of retrotransposon and SARS-CoV-2 RNA for potential human genome invasion of viral fragments, with the front and the rear part of SARS-CoV-2 genome being easier to form chimeric RNA. Thus, we suggest that primers and probes for nucleic acid detection should be designed in the middle of virus genome to identify live virus with higher probability. In summary, we propose our hypothesis that coronavirus invades human cells and interacts with retrotransposon, eliciting more severe symptoms in patients with underlying diseases. In the treatment of patients with coronavirus infection, it may be necessary to pay more attention to the potential harm contributed by retrotransposon dysregulation.

## Introduction

Emerging coronaviruses often spread rapidly from person to person and there seems to be an increased global outbreak of related diseases. MERS-CoV and SARS-CoV are two identified rare coronavirus strains which cause not only severe lung infection but also serious complications (Ksiazek et al., 2003; Rota et al., 2003; Zaki et al., 2012; Arabi et al., 2017). More recently, coronavirus disease named COVID-19 caused by a novel coronavirus SARS-CoV-2 is expanding globally and rapidly, resulting in emerging health issues (Chan et al., 2020; Guan et al., 2020; Huang et al., 2020; Zhou et al., 2020). Although cell receptors and the routes of infection of these coronaviruses have been identified (Li et al., 2003; Raj et al., 2013; Zhou et al., 2020; Wrapp et al., 2020), complicated impact on human cells is far from clear.

Transposable Elements (TEs) are mobile DNA elements in virtually all eukaryotes and comprise more than 40% of human genome (Dewannieux et al., 2003). They can self-replicate and insert into various locations inside genome. Dysregulation of TE may lead to various illnesses like inflammatory diseases (Saleh et al., 2019). The only active member in TE is retrotransposon which can “copy and paste” themselves through RNA intermediate. Examples of retrotransposons include Long interspersed nuclear elements (LINEs), short interspersed nuclear elements (SINEs) and long terminal repeats (LTRs). Expression of most of retrotransposon members is suppressed in somatic cells and they are only active in brains, germ cells, early embryos and pathological conditions (Munoz-Lopez et al., 2016). About 5% of newborn babies show a new retrotransposon integration event (Cordaux et al., 2006). Abnormally upregulation of retrotransposons cause insertions, deletions, and inversions in genome (Gilbert et al., 2002; Symer et al., 2002), resulting in compromised genetic stability and even cell death (Malki et al., 2014; Newkirk et al., 2017). Accumulated evidence in recent years also proved their importance in orchestration of gene expression (Izsvak et al., 2016), regulation of chromatin structure (Fadloun et al., 2013) and modulation of developmental program (Percharde et al., 2018; Lu et al., 2020).

LINEs are common autonomous retrotransposons and comprise about 17% of human genome (Cordaux and Batzer, 2009). Some LINE-1 elements can be transcribed and translated in cells. After reverse transcription of LINE-1 RNA, they can be integrated back into genome (Babushok et al., 2006). Naturally, LINEs expression is repressed in most cell types. Its RNA is mainly heritable during early embryogenesis because of its enrichment and high retrotransposition activity in early embryos (Grow et al., 2015). Transgenic mouse model carrying mouse/human LINE-1 retrotransposition reporter demonstrated that this activity creates somatic mosaicism during development (Kano et al., 2009). Besides LINEs, SINEs and LTRs are also enriched retrotransposons in human genome, and mobilization of SINEs relies on LINE-1-encoded proteins (Dewannieux et al., 2003).

In our study, we analyzed publicly available transcriptome data of human cells infected with coronavirus MERS-CoV, SARS-CoV, and SARS-CoV-2, and observed enhanced expression of TEs including several retrotransposons, as well as inflammation, immunity, and apoptosis related genes. We further noticed potential fusion of SARS-CoV-2 RNA with retrotransposon transcripts especially LINEs and SINEs. Therefore, further examinations on genome and transcriptome of cells from patients and studying models will be valuable to evaluate potential crosstalk between coronavirus and retrotransposons.

## Methods

### Cell Types Used for Transcriptome Study of Coronavirus Infection

Cell types below are used in this study. Calu-3, human lung cancer cell; MRC5, human fetal lung strain; A549, human adenocarcinomic alveolar basal epithelial cell; NHBE, primary human bronchial epithelial cell. Each group above has three replicates. For human intestinal organoids, each group has two replicates.

### RNA-Seq Data Processing

Raw reads were processed with cutadapt v1.16 to perform quality trimming with default parameters except for: quality-cutoff =20, pair-filter=both. To include as many non-uniquely mapped reads as possible, trimmed reads were firstly aligned to human/mouse genome (hg19/mm10) by STAR (v2.5.1b) with default settings including parameters “–outFilterMismatchNmax 10 –winAnchorMultimapmax 2000 –outFilterMultimapNmax 1000”. RSEM was used to calculate FPKM value of genes. The annotation and fasta sequences for consensus transposable element sequences were downloaded from Repbase (version 20.01) (Bao et al., 2015). TEtranscripts program (Jin et al., 2015) with default parameters was used to get counts for transposable elements. Read counts of gene and TE transcripts were normalized by total aligned counts. For RNA-seq alignment of coronavirus genomes, MERS-CoV (NC_019843), SARS-CoV (NC_004718) and SARS-CoV-2 (NC_045512) genomes were downloaded from NCBI, and trimmed reads were aligned to coronavirus genome by STAR (v2.5.1b) using default parameters. To identify potential chimeric transcripts of coronavirus and cellular transcripts from single-end RNA-seq data, 30nt fastq reads from each end were extracted from raw fastq reads and both were aligned to human and SARS-CoV-2 genomes respectively. Non-viral end of the chimeric reads were mapped to consensus transposable element sequences using STAR with parameters “–winAnchorMultimapmax 2000 –outFilterMultimapNmax 1000” to get counts of transposons. Integrative Genomics Viewer (IGV) and UCSC Genome Browser was used for snapshot of transcriptome. R package Deseq2 was used to get differential expressed genes. Metascape was used to visualize functional profiles of genes and gene clusters (Zhou et al., 2019). Graphs were created by R or Excel. Images were organized by Adobe Illustrator.

### Accession Number

RNA sequencing data of coronavirus-infected human lung-derived cells are from GSE122876 (transcriptome of MERS-CoV-infected Calu-3 cells; single-read; MOI 2, treated for 24 h) (Yuan et al., 2019), GSE56192 (transcriptome of MERS-CoV and SARS-CoV infected MRC5 cells; paired-end; MOI 2, treated for 24 h), GSE147507 (transcriptome of SARS-CoV-2 infected A549 cells, Calu-3 cells, and NHBE cells; MOI 2, treated for 24 h) (Blanco-Melo et al., 2020). RNA sequencing data of SARS-CoV-2-infected human intestinal organoids are from GSE149312 (MOI 1, treated for 24 and 60 h, grown in differentiation medium) (Lamers et al., 2020). SARS-CoV-2 infected Calu-3 cells were used to identify chimeric transcripts of coronavirus and cellular RNA. RNA sequencing data of IRF1 knockout and control human hepatocytes infected with hepatitis A virus are from GSE114916. RNA sequencing data of STAT1 knockout and control human HepG2 cells treated by IFN are from GSE98372 (Chen et al., 2017). RNA sequencing data of human tissues and cell types are from GSE83115 (Zhu et al., 2016). RNA sequencing data of human early embryos and embryonic stem cells are from GSE36552 (Yan et al., 2013). RNA sequencing data of 8-cell mouse embryos and adult mouse islet developed from zygotes with injection of sperm tsRNAs from high-fat-diet males are from GSE75544 (Chen et al., 2016).

## Results and Discussion

### Coronavirus Infection Disturbs Diverse Biological Processes in Human Cells and Can Stimulate ACE2 Expression Through IRF1 and STAT1

Coronaviral infection led to not only respiratory failure but also multiple organ dysfunction syndromes, indicating that coronavirus impacts a wide range of human cells (Wang et al., 2020). Transcriptome analysis may provide valuable information on how human cells react with coronavirus entry.

**Figure 1:**
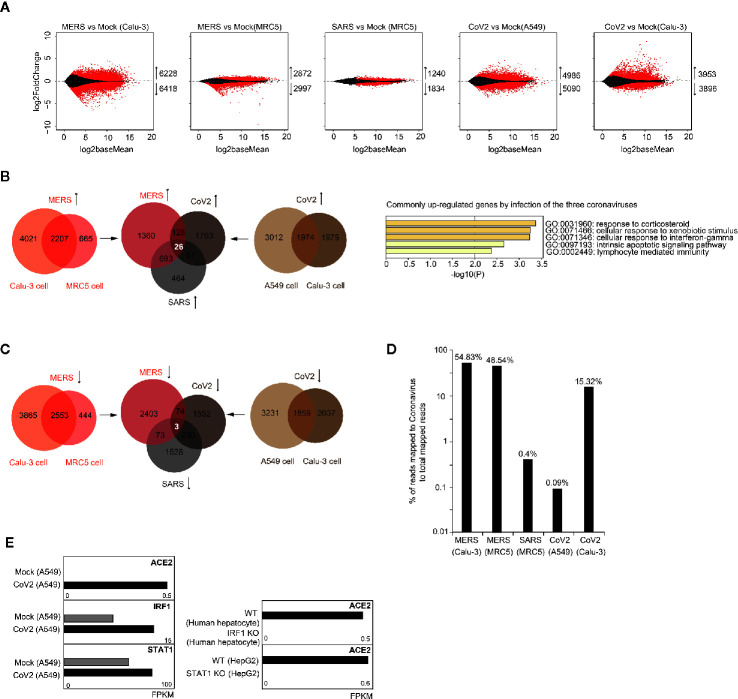
Analysis of transcriptome alteration induced by infection of various coronaviruses. **(A)** MA plot (log ratio RNA abundance versus log abundance) of RNA-seq data comparing control and coronavirus-infected cells. Differentially expressed genes with adjusted P<0.05 are highlighted in red. Numbers of up/down-regulated genes are indicated. Calu-3, MRC5, and A549 are all human cells with lung origin. MERS, MERS-CoV; SARS, SARS-CoV; CoV2, SARS-CoV-2. **(B)** Venn diagrams document 26 commonly upregulated genes by different coronavirus infection (left panel). Gene ontology analysis of the 26 genes for enriched biological processes (right panel). **(C)** Venn diagrams document only three commonly downregulated genes by SARS-CoV-2 infection in two cell types. **(D)** Bar graph indicates percentage of reads mapped to coronavirus genome to total mapped reads in human cells infected with coronavirus. **(E)** Bar graphs demonstrate that SARS-CoV-2 infection caused change of ACE2 expression from below detection to low level in A549 cells. SARS-CoV-2 infection also caused upregulation of IRF1 and STAT1. IRF1 knockout in human hepatocytes infected with hepatitis A virus decreased ACE2 expression. STAT1 knockout in IFN-treated human HepG2 cells decreased ACE2 expression.

To examine whether coronavirus infection disturbs expression of specific gene sets in human cells, we analyzed public available RNA-seq data of human lung-derived cells with infection of MERS-CoV, SARS-CoV, and SARS-CoV-2. Through comparison of transcriptomes before and after infection, we identified thousands of dysregulated genes (adjusted p-value < 0.05) for each group (**Figure 1A**). Among those dysregulated genes, we found that 26 genes were commonly upregulated after infection of the three coronaviruses (**Figure 1B**), but very few genes were identified to be commonly downregulated (**Figure 1C**). GO analysis of the 26 commonly upregulated genes demonstrated enrichment on inflammation, immunity and apoptosis related pathways (**Figure 1B**). Through relative viral sequence content in transcriptome, we found that the three coronaviruses can infect various human lung-derived cells (**Figure 1D**), however, low dose of coronavirus or using NHBE cells for infection were not successful to support coronavirus replication (**Figure S1**).

ACE2 is the cell receptor of SARS-CoV-2 (Zhou et al., 2020; Wrapp et al., 2020). Differently from robust expression of ACE2 in Calu-3 cells, ACE2 expression was undetectable in A549 cells, but after SARS-CoV-2 infection, low level of ACE2 was observed (**Figure 1E**). This indicates that transcription factors responding to coronavirus infection induced ACE2 expression. Recent report showed that ACE2 can be stimulated by interferon, and proposed IRF1 and STAT1-binding sites near ACE2 transcription start site (**Figure S2**) (Ziegler et al., 2020). Here, we noticed that expression of both IRF1 and STAT1 were increased after SARS-CoV-2 infection, and ACE2 expression was reduced when IRF1 was depleted in virus-infected human cells or STAT1 was depleted in interferon-treated human cells (**Figure 1E**). These results confirmed that IRF1 and STAT1 are essential upstream activators of ACE2 upon virus infection. So, we propose that SARS-CoV-2 might enter human cells with low efficiency by bulk-phase endocytosis in A549 cells, inducing IRF1 and STAT1 expression which further enhances ACE2 expression to facilitate receptor-mediated viral entry.

### Coronavirus Infection Enhanced Retrotransposon Expression in Human Lung-Derived Cells

**Figure 2:**
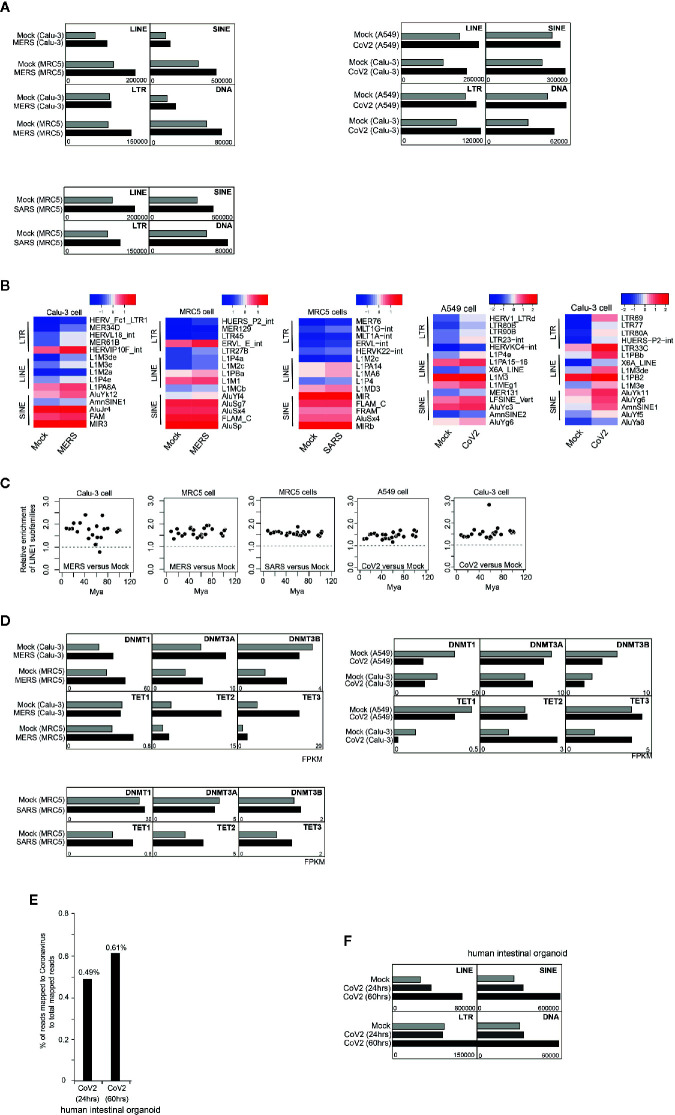
Coronavirus infection in human cells enhanced retrotransposon expression. **(A)** Bar graphs show that after 24-h MERS-CoV/SARS-CoV/SARS-CoV-2 infection in Calu-3/MRC5/A549 cells, expression of TE was generally increased. **(B)** Heatmap indicates upregulation of several LTR, LINE, and SINE elements induced by MERS-CoV/SARS-CoV/SARS-CoV-2 infection. Only top 5 upregulated transposable element (TE) subfamilies are shown. **(C)** Ratio of LINE1 upregulation was not determined by evolutionary age of LINE1 elements. Evolutionary age was calculated based on a substitution rate of 0.17%/million years. Mya, million years ago. **(D)** Bar graphs depict expression of genes encoding enzymes controlling DNA methylation status before and after MERS-CoV/SARS-CoV/SARS-CoV-2 infection in Calu-3/MRC5/A549 cells. **(E)** Bar graphs show that 24 h or 60 h post SARS-CoV-2 infection in human intestinal organoid, expression of TE was generally increased. **(F)** Bar graph indicates percentage of reads mapped to SARS-CoV-2 genome to total mapped reads in transcriptome of human intestinal organoids infected with SARS-CoV-2.

Next, we ask whether TE expression is impacted by coronavirus infection. We first examined transcriptome of human lung adenocarcinoma cell line Calu-3 after 24-h infection of MERS-CoV (Yuan et al., 2019). We observed that expression of TE including retrotransposons was generally activated after coronavirus infection (**Figure 2A**). Further examination documented that subfamilies of LINEs, SINEs, LTRs were differentially upregulated by coronavirus (**Figure 2B**). LINE-1 is the mostly well-studied autonomous retrotransposon. Most LINE-1 elements are inactivated in somatic cells, but some escape variously evolved silencing mechanisms. Hence, we ask whether evolutionarily old and young retrotransposons were impacted by coronavirus infection differently. We compared the ratio of fold change of specific LINE-1 element expression ordered by predicted evolutionary ages (Khan et al., 2006), and found that older and younger LINE-1 elements were similarly influenced (**Figure 2C**). One of the major mechanisms for LINE-1 silencing is DNA methylation, and we examined expression of genes encoding DNA methyltransferases (DNMTs) and Ten-eleven translocation (TET) enzymes mediating active DNA demethylation. We observed that Tet genes were generally upregulated after coronavirus infection (**Figure 2D**), and upregulated DNA demethylation activity may lead to demethylation of retrotransposon promoters. This result supports that increased retrotransposon expression was caused by genome-wide DNA demethylation. We obtained similar results in MERS-CoV/SARS-CoV infected MRC5 cells which are noncancerous human lung fibroblast cells (**Figures 2A–D**).

Recent COVID-19 outbreak is caused by the novel coronavirus SARS-CoV-2. Here, we explored transcriptomes of SARS-CoV-2 infected A549 and Calu-3 cells. Similar to MERS-CoV and SARS-CoV infection, we found general increase of multiple transposable elements (**Figures 2A, B**), no biased impact of older and younger LINE-1 elements by SARS-CoV-2 infection (**Figure 2C**). SARS-CoV-2 infection also causes upregulation of TET gene expression (**Figure 2D**). Similarly, SARS-CoV-2 was identified to have the capability of infecting human intestinal organoids (**Figure 2E**) and increased retrotransposon expression can also be observed post infection in a time-dependent manner (**Figure 2F**).

Therefore, upregulation of retrotransposon seems to be a common event induced by coronavirus infection, possibly through enhancing global DNA demethylation activity. Despite of similar upregulation of retrotransposon families triggered by the three coronaviruses, individual retrotransposons are differently dysregulated, and this may cause various phenotypes in human cells. Note that above results were from 24-h infection of coronaviruses, and impact of long-term infection should be more severe. Moreover, retrotransposon is able to encode proteins and can form retrovirus-like particles (Grow et al., 2015), so examination of coronavirus-infected samples may need to discriminate coronavirus from retrovirus-like particles because of upregulation of retrotransposons.

### Upregulation of Retrotransposon May Be Long-Term Memorized Epigenetically

We then ask whether retrotransposon upregulation can be long-term inherited through several generations of cell divisions. We found the mouse model of transgenerational epigenetic inheritance of acquired traits may provide molecular insights into this question.

**Figure 3:**
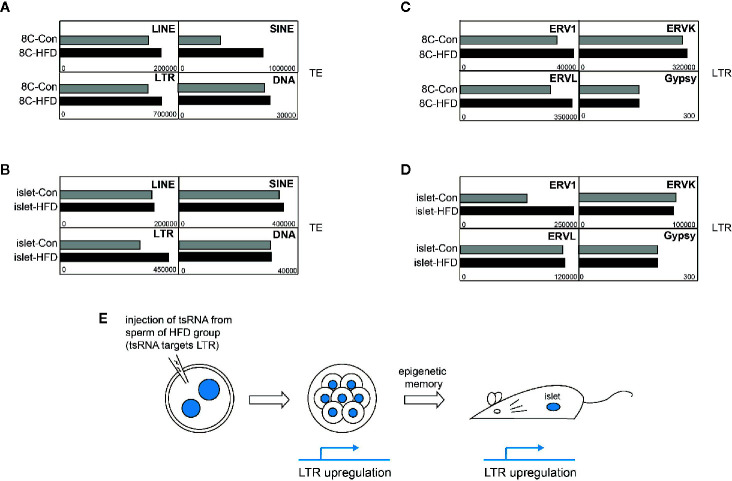
Potential long-term memory of transposable element (TE) upregulation. **(A)** Expression of TE subfamilies in 8-cell (8C) embryos developed from normal mouse zygote injected with tsRNA derived from sperm of high-fat-diet (HFD) male. **(B)** Expression of TE subfamilies in adult islet developed from normal mouse zygote injected with tsRNA derived from sperm of HFD male. **(C)** Expression of LTR elements in 8C embryos developed from normal mouse zygote injected with tsRNA derived from sperm of HFD male. **(D)** Expression of LTR elements in adult islet developed from normal mouse zygote injected with tsRNA derived from sperm of HFD male. **(E)** Scheme of the process from obtaining tsRNA injected zygote to examining TE expression in 8C embryos and adult islet.

tRNA-derived small RNAs (tsRNAs) in sperm were reported to transmit abnormal epigenetic information into preimplantation embryo, and epigenetic abnormality was further inherited to adult tissue, causing metabolic disorders (Chen et al., 2016). Two kinds of tsRNAs were previously identified to regulate retrotransposon LTR (Schorn et al., 2017), so we ask whether abnormal retrotransposon activity is inheritable during this process. We analyzed the transcriptome of cleavage mouse embryo and adult islet originated from zygote with injection of tsRNA of sperm from normal or high-fat diet (HFD) male mice. We found that LINE, SINE, and LTR retrotransposons were all upregulated in 8-cell embryo when HFD tsRNA was injected (**Figure 3A**). Notably, LTR retrotransposon also showed upregulation in adult islet (**Figure 3B**). Further analysis on LTR families supported that upregulation of ERV1 expression was inherited from early embryo (**Figure 3C**) to adult islet (**Figure 3D**), probably through DNA methylation inheritance at ERV1 locus. Therefore, above result indicates that enhancement of retrotransposon expression, ERV1 in this case, may be long-term inherited, even from cleavage-stage early embryos to adult tissues, with change of DNA methylation as the potential molecular mechanism (**Figure 3E**).

### SARS-CoV-2 RNA May Form Chimeric Transcripts With Retrotransposon RNA Especially LINE for Potential Insertion Into Host Genome

Coronaviruses are RNA viruses and are not supposed to integrate into host genome by themselves. However, it was reported that several RNA viruses have capacity to recombine with retrotransposons to invade host genome (Geuking et al., 2009). Regarding contribution of SARS-CoV-2 RNA to total transcriptome in infected Calu-3 cells to be as high as 15.32% (**Figure 1D**), we explored in the transcriptome the potential chimeric transcripts of SARS-CoV-2 and cellular RNA, and obtained subtranscriptome with chimeric reads.

**Figure 4:**
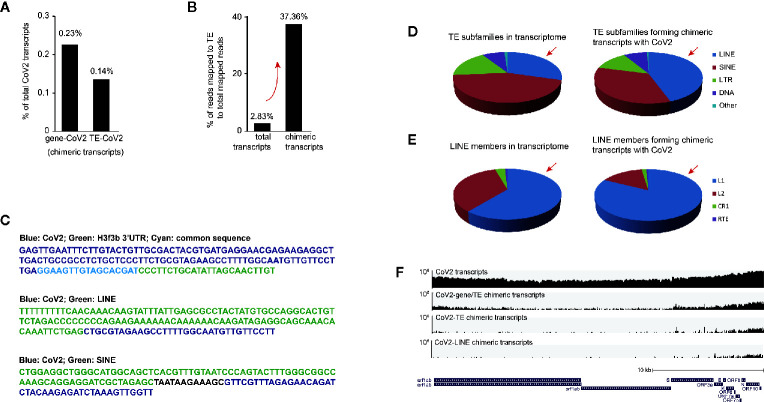
Retrotransposon-coronavirus chimeric transcripts were observed in SARS-CoV-2 infected human cells. **(A)** Bar graph shows relative enrichment of chimeric transcripts of coronavirus and cellular transcripts to total coronavirus transcripts. **(B)** Examination of ratio of mapped transposable element (TE) reads to non-TE gene reads in total transcriptome and in subtranscriptome of chimeric reads (between viral and cellular transcripts). **(C)** Example of chimeric reads with junctions of coronavirus-gene, coronavirus -LINE and coronavirus-SINE. **(D)** Pie charts demonstrate distribution of TE subfamilies in total transcripts (left panel) and coronavirus-retrotransposon chimeric transcripts (right panel) in SARS-CoV-2 infected Calu-3 cells. Red arrow indicates overrepresentation of LINE reads. **(E)** Pie charts demonstrate distribution of LINE members in total transcripts (left panel) and coronavirus-retrotransposon chimeric transcripts (right panel) in SARS-CoV-2 infected Calu-3 cells. Red arrow indicates overrepresentation of LINE-1 (L1) reads. **(F)** IGV snapshot of SARS-CoV-2 transcripts (upper first) and chimeric transcripts (between viral and cellular transcripts, viral and TE transcripts, as well as viral and LINE transcripts, lower three) identified in infected Calu-3 cells. SARS-CoV-2 genome was used for alignment. Logarithmic scale is displayed. The reference panel was obtained from UCSC genome browser.

We found that 0.23% of SARS-CoV-2 RNA formed chimeric transcripts with non-TE genes and 0.14% with TE (**Figure 4A**). Surprisingly, TE-virus chimeric reads contribute 37.36% to total mapped chimeric reads, while TE reads are only 2.83% in total mapped reads (**Figure 4B**), indicating that TE is much more efficient to form chimeric transcripts with SARS-CoV-2 RNA than non-TE genes. We randomly extracted reads from subtranscriptome of chimeric transcripts of SARS-CoV-2 and cellular RNA, and confirmed identity of the chimeric reads (**Figure 4C**).

We further analyzed distribution of TE subfamilies in total transcriptome and subtranscriptome with chimeric reads, and found that reads of retrotransposon LINE, SINE, and LTR were all enriched in the subtranscriptome of chimeric reads (**Figure 4D**). Unexpectedly, only LINE RNA was overrepresented in subtranscriptome with chimeric reads than in total transcriptome, and further analysis showed that virus-LINE-1 was overrepresented in virus-LINE reads (**Figure 4E**). This demonstrates high efficiency of LINE family especially LINE-1 in forming chimeric transcript with SARS-CoV-2 RNA. LINE-1 is autonomous retrotransposon with retrotransposition activity, and RNA-RNA ligation mediated by endogenous RNA ligase RtcB was previously reported for LINE-1 to carry other types of RNA for host genomic invasion (Moldovan et al., 2019), so similar mechanisms may apply for SARS-CoV-2 transcripts. Further examination of human genome from SARS-CoV-2 infected human cells or biopsies will be particularly important to identity existence of integration of coronavirus RNA into human genome.

Moreover, to identify which region of SARS-CoV-2 RNA prone to form chimeric transcripts with cellular RNA, we obtained subtranscriptome of chimeric transcripts, extracted SARS-CoV-2 reads, and aligned to SARS-CoV-2 genome, and viewed on IGV to find that the front and the rear parts, especially the rear part of coronavirus RNA were biased in forming chimeric transcripts (**Figure 4F**). Moreover, our further examination showed that only the rear part of SARS-CoV-2 is prone to form chimeric RNA with TE/LINE (**Figure 4F**). However, more direct evidence is needed to prove existence of chimeric transcripts and potential human genome integration, for example, through genome sequencing of blood cells from coronavirus-infected patients. Based on above analysis, we suggest that primers and probes for SARS-CoV-2 testing are designed in middle of the SARS-CoV-2 genome.

### Our Hypothesis on Coronavirus-Retrotransposon Interaction

**Figure 5:**
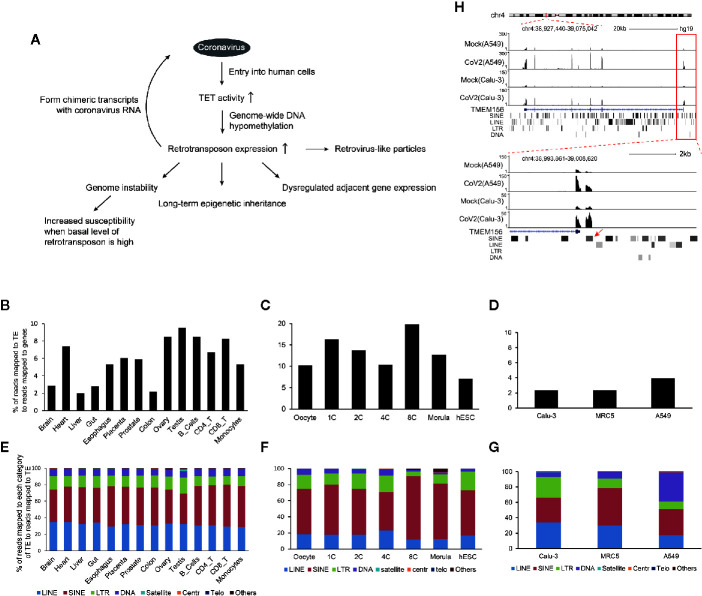
Model of how coronavirus may impact retrotransposons to harm human cells. **(A)** Generally, entry of coronavirus into human cells enhances ten-eleven translocation (TET) activity for genome-wide DNA hypomethylation to facilitate retrotransposon enhancement. This may lead to increased fusion between viral and retrotransposon transcripts, reduced genome stability, and increased susceptibility of aged people and cancer patients, dysregulated TE-adjacent gene expression, and this influence may be inherited for a long term. Meanwhile, increased retrovirus-like particles of retrotransposons may be induced. **(B–G)** Endogenous retrotransposon expression and distribution of subfamilies are variable in human tissues and cells. Bar graphs indicate percentages of reads mapped to TE to reads mapped to genes in human tissues and immunocytes **(B)**, human oocytes and early embryos **(C)**, and normal Calu-3, MRC5 and A549 cells **(D)**. Bar charts **(E**–**G)** demonstrate distribution of individual subfamilies of TE in tissues or cell types shown above. **(H)** UCSC genome browser view of an example of retrotransposon-initiated gene expression by readthrough mechanism.

Based on above analysis, we propose our hypothesis that coronavirus infection may increase retrotransposon expression through modulating TET activity to reduce global DNA methylation. Increased retrotransposon RNA may further form chimeric transcripts with coronavirus RNA, and integrate viral genomic fragments into human genome. Moreover, enforced retrotransposon expression may be harmful and probably long-term inherited (**Figure 5A**).

TE is widely expressed in human tissues (**Figure 5B**), with highest enrichment in early human embryos (**Figure 5C**). The cells used in this study are mainly derived from human lung and also robustly express TE (**Figure 5D**). Moreover, TE subfamilies are variable in different cell types (**Figures 5E–G**), suggesting extensive but specific phenotype upon global retrotransposon upregulation.

The first concern regarding global retrotransposon upregulation is genome instability. Retrotransposition activity is high in early embryo (Grow et al., 2015) and brain (Zhao et al., 2019) during normal development, so potential integration of coronavirus sequence into human genome is suggested to be scrutinized for these cells. It was also reported that retrotransposon upregulation is positively correlated with tumor progression (Jung et al., 2018), causing genomic deletion, translocation, and duplication (Rodriguez-Martin et al., 2020). What’s more, increased expression of retrotransposon LINE-1 contributes to age-associated inflammation in several tissues (De Cecco et al., 2019). Additionally, vapers and smokers demonstrated higher retrotransposon expression and hypomethylation at associated loci (Caliri et al., 2020). Also, people with neurological disorders may have higher retrotransposon expression and retrotransposition activity (Terry and Devine, 2019). These reports not only show that upregulation of retrotransposon expression may cause several diseases, but also indicate that persons with higher basal level of retrotransposons are supposed to be more susceptible to coronavirus infection and have increased risk of symptomatic infection. In support of this, recent analysis of SARS-CoV-2 patients showed that cancer patients (Liang et al., 2020) and aged people (Wu et al., 2020) get more severe symptoms after infection. Therefore, inhibition of reverse transcriptase activity in human cells may be necessary during pharmaceutical treatment of coronavirus-infected patients, especially those with higher basal level of retrotransposons.

The second concern regarding global retrotransposon upregulation is disturbance of retrotransposon adjacent gene expression. Accumulated evidence shows that retrotransposons are not just genomic fossils, but have molecular functions. For example, physically adjacent retrotransposon activates gene promoter of TMEM156 or MYADM by readthrough mechanism (**Figure 5H**, **Figures S3-S5**, **Table S1**) in both SARS-CoV-2 infected A549 and Calu-3 cells, and the read-through mechanism for BCL3 gene is shown in SARS-CoV-2 infected A549 cells (**Figure S6**). Also, transcripts of LINEs, SINEs and low-complexity repeats physically interacted with specific genomic areas to play distinct roles (Ding et al., 2004).

The third concern regarding global retrotransposon upregulation is whether coronavirus RNA can enter nucleus and associate with specific genomic regions through sequence homology, similar like the behavior of retrotransposon RNA (Ding et al., 2004; Fadloun et al., 2013). Blast analysis in NCBI using SARS-CoV-2 genome showed no similar sequence in human genome. We further used CENSOR program (Jurka, 1998) to analyze the SARS-CoV-2 genome and all predicted candidate repetitive elements are less than 200bp. Therefore, no evidence supports that SARS-CoV-2 RNA has the ability to recognize human genome by homologous sequence even these transcripts enter nucleus by chance.

## Conclusions

Taken together, we demonstrate that coronavirus infection increases retrotransposon expression in human cells, possibly through global DNA hypomethylation, and increased retrotransposon RNA may further form chimeric transcripts with coronavirus RNA for integration of viral genomic fragments into human genome. These enhanced retrotransposon transcripts may be long-term inherited to harm host organs. Therefore, we propose that retrotransposon upregulation induced by coronavirus infection may have potential contributions to coronavirus caused symptoms, and suggest careful transcriptome examination and genetic tests in future investigations on coronavirus-infected patients. Finally, we note that our hypothesis needs further validation in a more direct manner.

## Data Availability Statement

RNA sequencing data of MERS-CoV-infected Calu-3 cells (Zhou et al., 2019) are obtained from NCBI Sequence Read Archive with BioProject ID: PRJNA506733 (https://www.ncbi.nlm.nih.gov/bioproject/PRJNA506733/). RNA sequencing data of MERSCoV-infected and SARS-CoV-infected MRC5 cells are obtained from NCBI Sequence Read Archive with BioProject ID:PRJNA233943 (https://www.ncbi.nlm.nih.gov/bioproject/PRJNA233943). RNA sequencing data of SARS-CoV-2 infected A549 cells, Calu-3 cells, and NHBE cells (Yuan et al., 2019) are obtained from NCBI Sequence Read Archive with BioProject ID: PRJNA615032 (https://www.ncbi.nlm.nih.gov/bioproject/PRJNA615032). RNA sequencing data of SARS-CoV-2-infected human intestinal organoids (Blanco-Melo et al., 2020) are obtained from NCBI Sequence Read Archive with BioProject ID: PRJNA628628 (https://www.ncbi.nlm.nih.gov/bioproject/PRJNA628628/). RNA sequencing data of IRF1 knockout and control human hepatocytes infected with hepatitis A virus are are obtained from NCBI Sequence Read Archive with BioProject ID: PRJNA473130 (https://www.ncbi.nlm.nih.gov/bioproject/PRJNA473130/). RNA sequencing data of STAT1 knockout and control human HepG2 cells treated by IFN (Lamers et al., 2020) are obtained from NCBI Sequence Read Archive with BioProject ID: PRJNA384926 (https://www.ncbi.nlm.nih.gov/bioproject/PRJNA384926/). RNA sequencing data of human tissues and cell types (Chen et al., 2017) are obtained from NCBI Sequence Read Archive with BioProject ID: PRJNA324812 (https://www.ncbi.nlm.nih.gov/bioproject/PRJNA324812/). RNA sequencing data of human early embryos and embryonic stem cells (Zhu et al., 2016) are obtained from NCBI Sequence Read Archive with BioProject ID: PRJNA153427 (https://www.ncbi.nlm.nih.gov/bioproject/PRJNA153427/). RNA sequencing data of 8-cell mouse embryos and adult mouse islet developed from zygotes with injection of sperm tsRNAs from high-fat-diet males (Yan et al., 2013) are obtained from NCBI Sequence Read Archive with BioProject ID: PRJNA304514 (https://www.ncbi.nlm.nih.gov/bioproject/PRJNA304514/).

## Author Contributions

L-QZ and XH conceived and designed the project. YY analyzed the data and wrote the manuscript. X-ZL performed analysis on chimeric transcripts. L-QZ and XH revised the manuscript. All authors contributed to the article and approved the submitted version.

## Funding

This work was supported by the National Key R&D Program of China [2018YFC1004502, 2018YFC1004001] and the National Natural Science Foundation of China [NSFC 31771661, 32000488].

## Conflict of Interest

The authors declare that the research was conducted in the absence of any commercial or financial relationships that could be construed as a potential conflict of interest.

## Supplementary Material

Supplementary Figure 1 Related to **Figure 1**, viral dose and cell type influences SARS-CoV-2 replication. Bar graph indicates percentage of reads mapped to SARS-CoV-2 genome to total mapped reads of human cells infected with SARS-CoV-2.

Supplementary Figure 2 Related to **Figure 1**, potential binding sites of IRF1 and STAT1 at human ACE2. Scheme displays locations of potential genomic binding sites of IRF1 and STAT1 near transcription start site of human ACE2 gene.

Supplementary Figure 3 Related to **Figure 5H**, representative alignment near SINE-UTR junction at TMEM156 locus in SARS-CoV-2 infected A549 or Calu-3 cells.

Supplementary Figure 4 UCSC genome browser view of an example of retrotransposon-initiated MYADM gene expression by readthrough mechanism.

Supplementary Figure 5 Related to Figure S4, representative alignment near LTR-UTR junction at MYADM locus in SARS-CoV-2 infected A549 or Calu-3 cells.

Supplementary Figure 6 Representative alignment near SINE-UTR junction at BCL3 locus in SARS-CoV-2 infected A549 cells.

Supplementary Table 1 The ratio of read-through reads to total initiating TE reads for TMEM156, MYADM and BCL3 genes.

## Abbreviations

LINE, long interspersed nuclear element; LTR, long terminal repeat; SINE, short interspersed nuclear element; TE, transposable element.
