# Supplementary figures and images for: Exogenous Coronavirus Interacts With Endogenous Retrotransposon in Human Cells
Source: Front Cell Infect Microbiol. 2021 Feb 25;11:609160. doi: 10.3389/fcimb.2021.609160 (PMC7959850; doi:10.3389/fcimb.2021.609160)

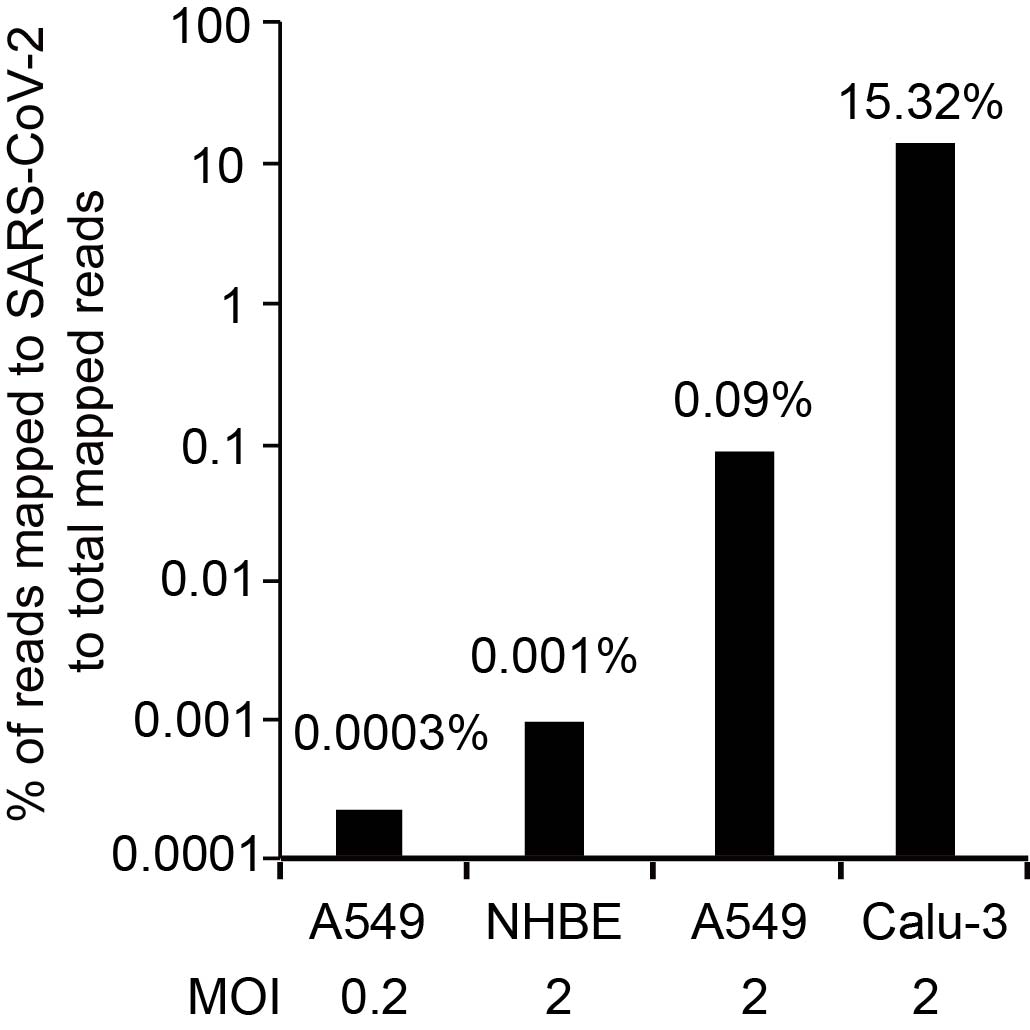

Supplement: Supplementary Figure 1 — Related to Figure 1 , viral dose and cell type influences SARS-CoV-2 replication. Bar graph indicates percentage of reads mapped to SARS-CoV-2 genome to total mapped reads of human cells infected with SARS-CoV-2. [file Image_1.jpeg]

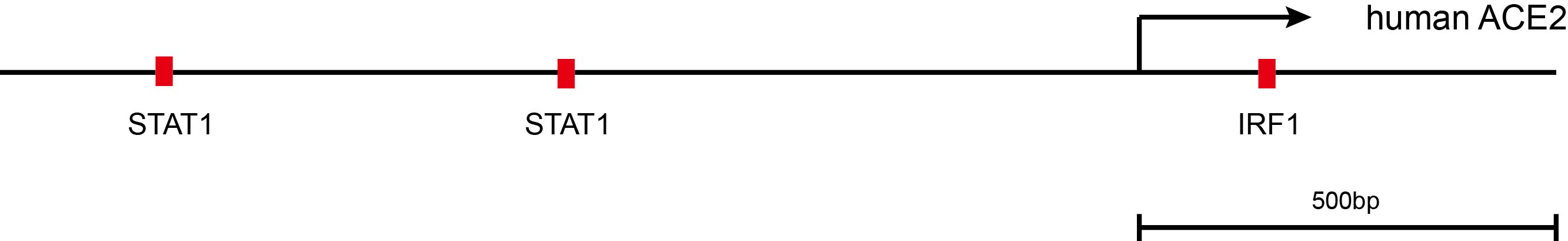

Supplement: Supplementary Figure 2 — Related to Figure 1 , potential binding sites of IRF1 and STAT1 at human ACE2. Scheme displays locations of potential genomic binding sites of IRF1 and STAT1 near transcription start site of human ACE2 gene. [file Image_2.jpeg]

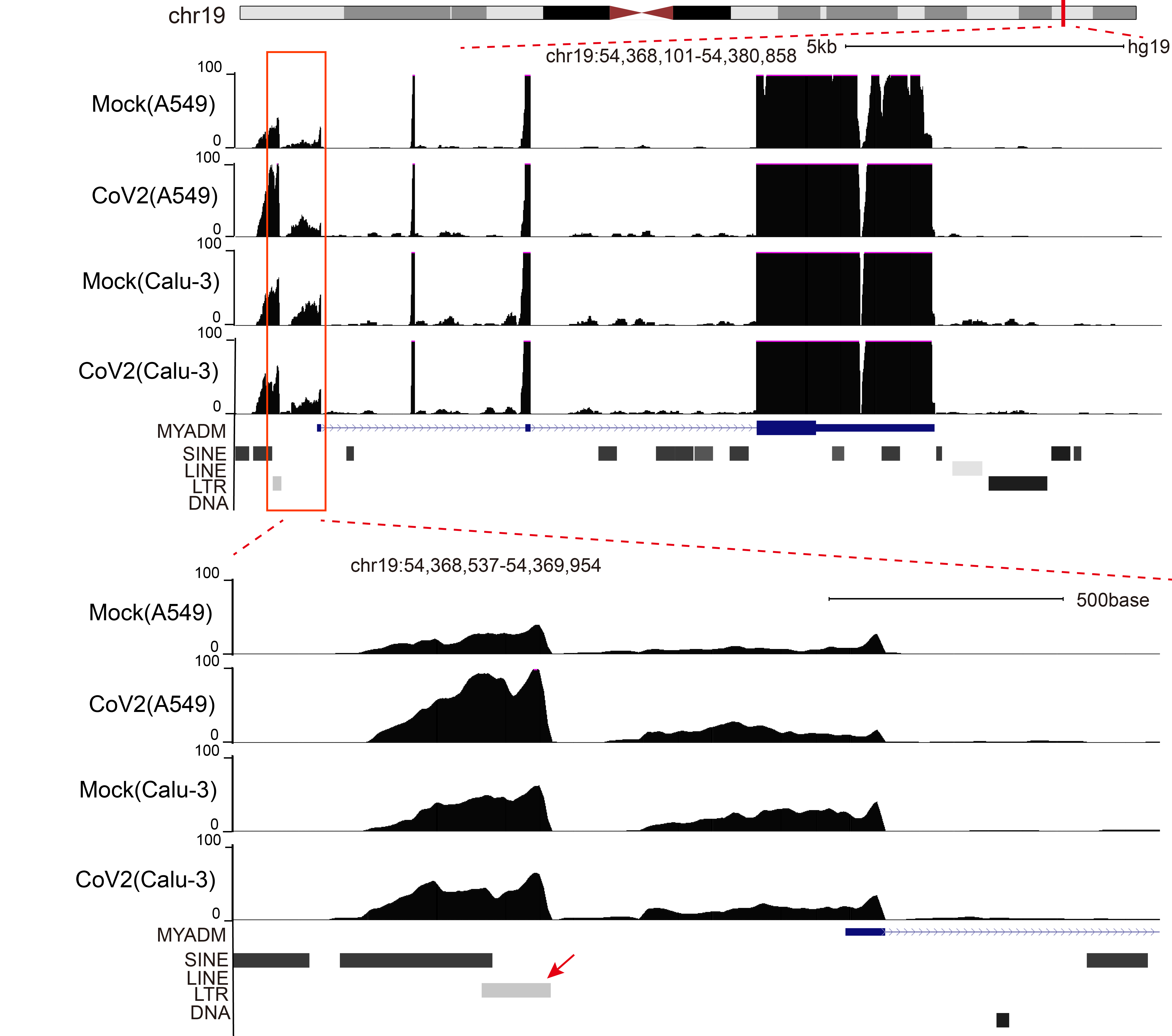

Supplement: Supplementary Figure 4 — UCSC genome browser view of an example of retrotransposon-initiated MYADM gene expression by readthrough mechanism. [file Image_4.jpeg]

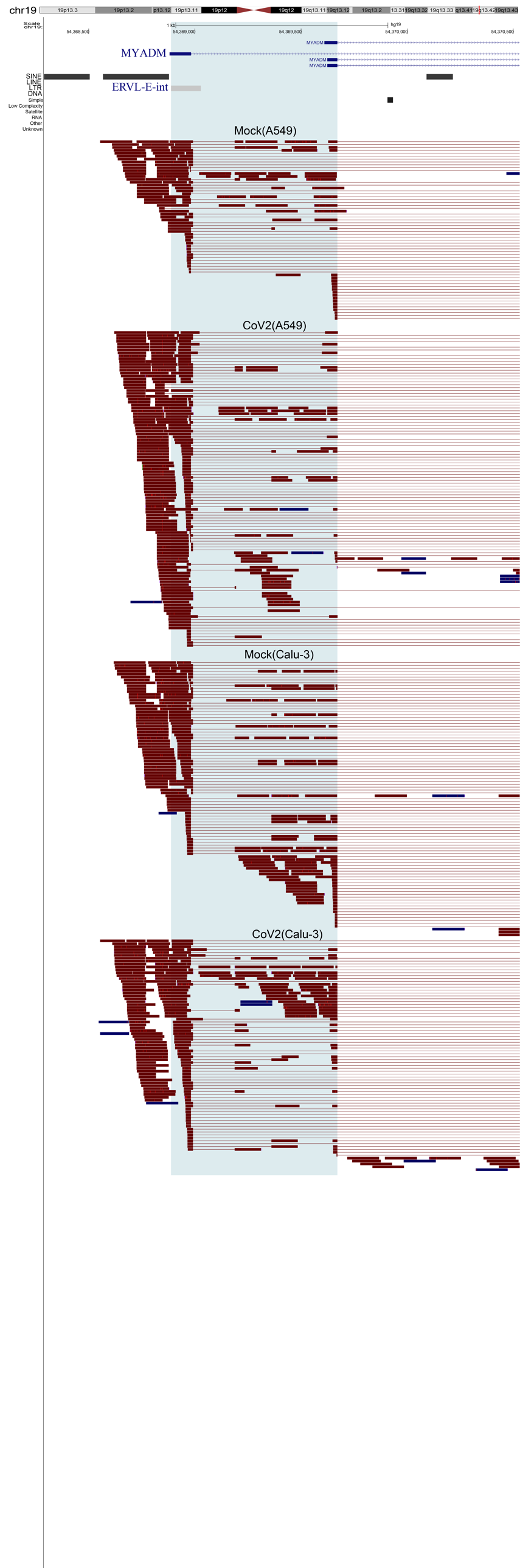

Supplement: Supplementary Figure 5 — Related to Figure S4, representative alignment near LTR-UTR junction at MYADM locus in SARS-CoV-2 infected A549 or Calu-3 cells. [file Image_5.jpeg]
